# The Effects of Internal Attention on Knee Biomechanics in Volleyball Spike Jump through Augmented Video Feedback

**DOI:** 10.3390/brainsci11050541

**Published:** 2021-04-25

**Authors:** Aiwen Wang, Xiaohan Li, Huiming Huang

**Affiliations:** Faculty of Sport Science, Research Academy of Grand Health, Ningbo University, Ningbo 315211, China; wangaiwen@nbu.edu.cn (A.W.); 2011042027@nbu.edu.cn (X.L.)

**Keywords:** ACH theory, volleyball, biofeedback, spike jump, internal attention

## Abstract

Poor knee biomechanics in a volleyball spike jump generally result in a higher knee injury risk, which can be altered by an internal focus of attention (FOA). The constrained action hypothesis (CAH) purports that the FOA inhibits sports performance whereas no ecologically valid evidence has been found in previous studies. The purpose of this research is to explore the effect of video feedback on knee biomechanics in a volleyball spike jump including landing and take-off phases. The video feedback was performed in a natural way. Fourteen volleyball male players were recruited in this study. A paired t-test was used to detect the effect of the feedback; meanwhile, statistical parameter mapping (SPM) statistics were used for the continuum differences during movement. After biofeedback, the initial contact flexion angle of the knee (*t* = 2.179, *p* = 0.049), the maximal flexion angle of the knee (*t* = 3.242, *p* = 0.006) and the maximal internal rotation angular velocity of the knee (*t* = 5.209, *p* = 0.003) increased significantly; the maximal extension moment of the knee (*t* = 3.962, *p* < 0.001) and the maximal flexion moment of the knee (*t* = −3.711, *p* = 0.002) significantly decreased; the maximal abduction moment significantly decreased (*t* = 3.069, *p* = 0.037) but the maximal internal rotation moment significantly increased (*t* = 2.813, *p* = 0.018); the first peak of the vertical ground reaction force (vGRF) (*t* = 7.618, *p* < 0.001) and the average loading rate to the first peak (*t* = 4.205, *p* = 0.004) significantly decreased; the other peaks of the vGRF were not found to have differences; a larger knee flexion was found during the phase from 31.17 to 73.19% (*t* = 2.611, *p* = 0.012); a larger adduction angular velocity was found during the phase from 49.07 to 62.46% (*t* = 3.148, *p* = 0.004); a smaller external rotational angular velocity was found during the phase from 45.85 to 49.96% (*t* = 5.011 *p* = 0.017); there was an increased flexion moment of the knee during the phase from 19.72 to 21.38% (*t* = 0.029, *p* = 0.029) and an external moment of the knee during the phase from 85.55 to 95.06% (*t* = 4.214, *p* < 0.001); the vGRF significantly decreased during the phase from 3.13 to 5.94% (*t* = 4.096, *p* = 0.014) and 19.83–21.97% (*t* = 4.096, *p* = 0.024) but significantly increased in the phase of 91.43–100% (*t* = 4.096, *p* < 0.001). The impulse of the vGRF and knee power were not found to be different compared with before biofeedback. Therefore, our study suggests video feedback in a natural practice has the potential to improve knee movement whilst not altering the performance in a volleyball spike jump. This indicates that the CAH theory is possibly not suitable in a real competition. Due to the complexity of human movements and the limitations of this study, muscle activities must be considered in the future.

## 1. Introduction

Players are required to perform multiple technically complex movements in volleyball competitions. Research studies have shown that sports that require cutting, stop-jumps, landing or sudden deceleration before changing direction usually result in acute injuries to the knee [1,2,3] including a Meniscus tear and non-contact anterior cruciate ligament tears [4,5]. The spike jump (SPJ) is one of the most important skills in determining tactical success in volleyball, which can be improved by biofeedback [6,7]. The adverse biomechanics of the lower limb during an SPJ increases knee injury risk in volleyball [2] and poor landing biomechanics of lower limbs especially link to an anterior cruciate ligament (ACL) or patella-femoral joint (PFJ) injury [8,9]. To our knowledge, there is no effective training protocol or guidelines that can improve poor biomechanics in volleyball.

Specific motor patterns are related to the psychological status; for example, short- and long-term memories that alter attention strategies [10]. An attentional strategy can affect movement execution that results in different consequences [11,12]. Apart from inadequate muscle strength of the gluteus medius, gluteus maximus and quadriceps [13] and poor proprioception [14] and limited lower limb joints resulting in compensatory movement patterns [15], lower limb injuries perhaps result from the simplest cause of a lack of attention. The individual’s focus of attention (FOA) plays a vital role in performing a complex motor skill [16]. The FOA can be classified into internal or external focus in the literature. An internal FOA is directed towards bodily moments whereas an external FOA is directed to the effect of the movement. The constrained action hypothesis (CAH) suggests that internal FOA makes individuals control the process more actively [15] yet it inhibits the performance in motor skills. The CAH is proposed according to a complicated protocol including inappropriate verbal or video instructions in labs [17], which is quite different from real practice and cannot represent the reaction in a real condition [18]. Previous studies cannot provide an ecologically valid evidence of the CAH. The relationship between internal FOA and performance in a real sports setting is still unknown. For instance, a clue that instructs the player to focus on the arm motion throughout golf practice would interrupt the automatic process but it cannot represent the process in real practice [18]. Players can switch their focus between internal FOA and external FOA automatically; the process of which is controlled by the players according to the real-time conditions [19]. The pursuit for performance in training and the increase of injury risk are generally considered to be conflicting [20] but it is possible for players to obtain both performance and lower injury risk in a real competition through achieving a balance between internal FOA and external FOA based on biofeedback. Hence, whether video feedback inhibits sports performance in a real condition needs to be identified.

Video augmented feedback is indicated to prevent knee injuries and improve skill development [21,22]. Video-related technologies are widely applied in sports nowadays, which provides an opportunity to use video feedback in competitions. Compelling evidence has proven the use of video feedback to be a vital part associated with optimizing lower limb movements [16,21]. Even if the reduction of the peak of the vGRF and the improvement of kinematics during a spike jump have been reported frequently in literature [16,21], few knee kinetics have been reported as to the effect of video feedback on knee movement. Although it is hard to explore the effect of video feedback on knee biomechanics in a sport-specific situation, exploring in a natural practice can provide an important vision not only on the effect of video feedback on knee biomechanics but also on ACH theory.

A discrete value analysis has been conventionally used whereas the single value cannot represent the curve for ignoring the time characteristics throughout the entire emergency take-off or landing phase [23]. Statistical Parametric Mapping (SPM) has the advantage of detecting the curve differences of continuous data such as angles and the moment, which are considered to represent the signal differences with time changing [24]. Hence, both conventional and SPM methods were adopted in our study to further examine the effects of video feedback on the knee.

This study aims to examine the effect of video feedback on knee biomechanics in a natural practice. We hypothesized that the video feedback in a natural way would improve knee biomechanics without inhibiting performance during an SPJ in volleyball.

## 2. Materials and Methods

### 2.1. Participants

Fourteen healthy male college volleyball players (age 20.4 ± 0.81 years; height 186.8 ± 1.39 cm; mass 77.4 ± 3.27 kg) were recruited for this study at Ningbo University. All participants were right spikers and played at the fourth position. They signed an informed consent approved by the University’s Institutional Review Board before study participation. The eligible criteria were (1) non-history of lower extremity disease or surgery; (2) free from sports injuries or no medical problems within six months of the start of the experiment. All participants had not joined similar tests without video feedback being given before.

### 2.2. Testing Procedure

**Figure 1:**
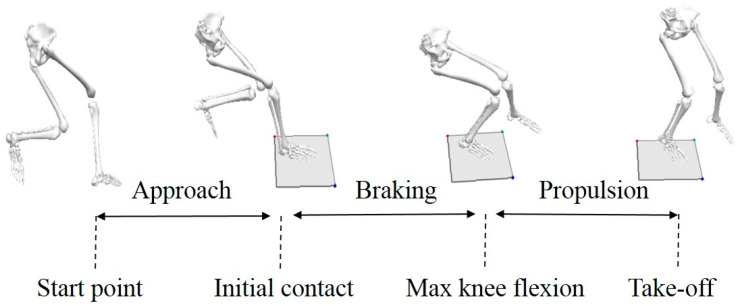
The process of a jump following a spike.

Basic information including height, weight, age and the dominant leg was collected before the completion of the biomechanics assessment. The dominant limb was defined as the limb used to kick a soccer ball. A Vicon System consisting of 8 infrared high-speed cameras (200 Hz, Oxford Metric Ltd., Oxford, UK) was used for the data collection of biomechanics, which was synchronized with a force platform (1000 Hz, AMTI, Watertown, MA, USA). The infrared high-speed camera was set up around the force measuring platform with a net (2.43 m) placed in the testing area; the distance from the net to the platform was a half meter. After the warm-up procedure (8 min of light jogging and hitting a volleyball back and forth between partners), the participants performed multiple test trials of the spike in the fourth position for familiarization with the ball setting from the optimal position. Trials were collected before and after the video feedback for each subject and a 30 min break was set before the video feedback to prevent fatigue. The following criteria were required: (1) right foot contacted the force platform on the ground and the other foot was outside the force platform; (2) both the player and the coach agreed that a maximal jump height and powerful strike were achieved; (3) the ball’s landing point was within the designated area and five qualified trials were recorded for each condition. The spike jump is illustrated with the definitions of the continuous phases in Figure 1. In addition to the biomechanics test, a maximal height test of a spike jump was performed to obtain the maximal jump height of players.

### 2.3. Video Feedback Protocol

The video content was about a standard spike jump performed by an elite player. The video contained two parts with the first part one minute of normal speed video and the second part two minutes of half normal speed video [7]. To simulate the real practice, we did not give the subjects any cues about the video content when subjects watched the video. The participant had to watch the given video once alone with the experimenters 3 m away. After the video feedback finished, participants were instructed to focus neither on their body movement nor on the ball deliberately and play as usual in the coming testing.

### 2.4. Data Processing

A total of 20 reflex markers with a diameter of 12.5 mm were attached to the subjects’ dominant limb and pelvis referring to a previously described marker set [25]. The standing trial used for the relative computation was captured before the formal test with an anatomically neutral posture. The trajectories and force platform signals were filtered using fourth order Butterworth low-pass filters with cut-off frequencies of 7 Hz and 15 Hz [26], respectively. The take-off phase was defined from the previous frame at which the heel touched on the force platform to the next frame at which the toe left off the force platform, which was detected by a threshold of the vGRF of 10 N. A 6-freedom rigid body model of bilateral lower limbs was created for the biomechanics computation in Visual 3D (C-Motion, Bethesda, MD, USA). The aimed kinematic and kinetic variables were calculated based on Visual 3D (C-Motion, Bethesda, Maryland, USA) then were transferred into MATLAB for further calculations. The jump height was calculated for each trial to examine whether the maximal height was achieved by comparing the height with the result obtained in the maximal height test (within a maximal height ± 95% CI). All kinematics and kinetics data were calculated using the Euler sequence X-Y-Z [27] with a positive value indicating knee extension, abduction and external rotation and a negative value indicating knee flexion, adduction and internal rotation. The approaching velocity was the resultant velocity relative to the ground when the right foot initially touched the force plate. The vGRF was normalized by body weight and joint movement and the power was normalized by body mass.

### 2.5. Statistical Analysis

Both discrete and SPM statistical techniques were used to detect the video feedback’s effect. The descriptive data were presented as a mean and standard deviation (SD). A paired t-test of discrete variables was performed for knee kinematics including angles at the initial contact and maximal and minimum angles in all three planes. Knee kinetics data referred to peaks of power, moment and the vGRF with loading rate of the vGRF entered in SPSS 24.0 (SPSS Inc, Chicago, IL, USA). The Shapiro–Wilk normality test was first used to check the assumption of the Gaussian distribution. If the data did not conform to the normal distribution, the Wilcoxon paired sign rank test was performed.

The SPM analysis was used for the detection of the regional differences in all kinematic and kinetic signals including knee angles, angular velocities, knee moment and power. All kinematic and kinetic data of the take-off phase were extracted and normalized to 101 data points (representing 0–100% of the take-off phase) with a custom MATLAB script. The paired t-test script within the SPM1D method was used in the current study [28]. In all tests, α was set to 0.05 to determine the differences.

## 3. Results

**Figure 2:**
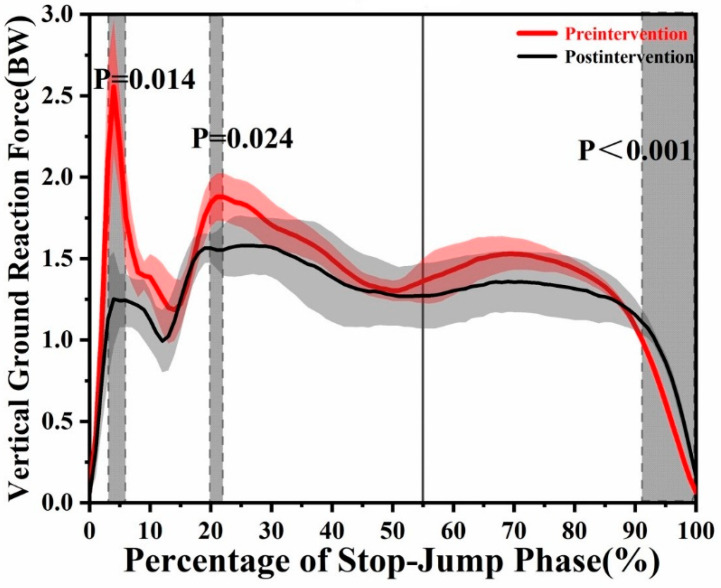
The results of statistical parametric mapping (SPM) pre- and post-video feedback depicting the mean of the vertical ground reaction force (vGRF). The solid line at 55% represents the time of maximum knee flexion. The grey region indicates a statistical difference (*p* < 0.05).

**Figure 3:**
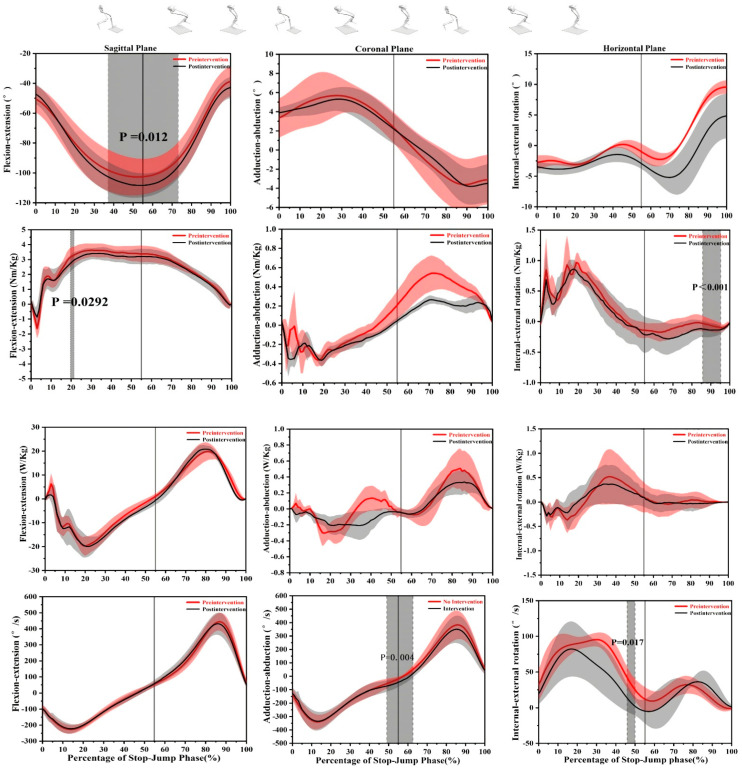
The results of statistical parametric mapping (SPM) pre- and post-video feedback depicting the mean angle, moment, power and angular velocity with a standard error in the knee sagittal, coronal and transverse planes. The solid line at 55% represents the time of maximum knee flexion. The grey region indicates a statistical difference (*p* < 0.05).

The temporal patterns of kinematics and kinetics variables in the sagittal, coronal and transverse planes of knee movements during an SPJ were evaluated using the SPM method (Figure 2 and Figure 3). The results of the discrete values are listed in Table 1 and Table 2.

A larger knee flexion angle was found after feedback during the phase from 31.17 to 73.19% (*t* = 2.611, *p* = 0.012). A larger adduction angular velocity was found after intervention between 49.07 to 62.64% (*t* = 3.148, *p* = 0.004) while there was no difference in other parameters. The results in the transverse plane showed that the external rotation angular velocity after video feedback decreased significantly from 45.85 to 49.96% (*t* = 5.199, *p* = 0.017) (Figure 3). Discrete kinematic variables in the sagittal, coronal and transverse planes of the knee are listed in Table 2. The contact knee flexion angle (*t* = 2.169, *p* = 0.049), the maximal knee flexion angle (*t* = 3.242, *p* = 0.006) and maximal internal angular velocity (*t* = 5.209, *p* = 0.003) increased significantly after video feedback while the other discrete variables did not show differences.

There was a significant increase of the flexion moment from 19.72 to 21.38% (*t* = 0.029, *p* = 0.029) and the external moment from 85.55 to 95.06% (*t* = 4.214, *p* < 0.001) after video feedback (Figure 3). There was no significant difference in the kinetics variables in the coronal plane. The vGRF was significantly decreased from 3.13 to 5.94% (*t* = 4.096, *p* = 0.014) and 19.83–21.97% (*t* = 4.096, *p* = 0.024); in contrast, it significantly increased in the phase of 91.43–100% (*t* = 4.096, *p* < 0.001) after intervention (Figure 2). Table 2 shows that the peak of extension moment (*t* = 3.962, *p* < 0.001) and the maximal flexion moment (*t* = 3.711, *p* = 0.002) were also significantly decreased. The maximal abduction moment significantly reduced (*t* = 3.069, *p* = 0.037) while the maximal internal rotation moment significantly increased (*t* = 2.813, *p* = 0.018) as well. The first peak of the vGRF (*t* = 7.638, *p* < 0.001) and the average loading rate to the first peak (*t* = 4.225, *p* = 0.004) were significantly higher before video feedback. There was no significant difference in the other parameters (Table 1).

## 4. Discussion

Motor control involves the integration of multiple sources of sensory feedback with feed-forward motor commands at multiple levels in the central nervous system [29]. Movement can be designed, modified and governed by the central nervous system. According to the CAH theory, when players use an internal focus of attention (focus on their movements), automatic control processes are interrupted [15] and then sports performance is undermined. However, in real practice players can freely switch their attention, which cannot be controlled [19]. Obtaining performance as well as a lower injury risk through the proper transition from an internal to external focus is possible for players.

The findings of the current study supported our hypothesis that video feedback in a natural way decreased the knee injury risk without inhibiting the performance. The results showed that there was no significant difference in the approaching velocity between before and after video feedback; thus, variations caused by the different approaching velocities could be excluded. This study showed that video feedback did not alter the impulse of the vGRF but decreased the vGRF in a few regions, which was consistent with previous studies [6,30]. The finding in this study that video feedback did not alter the impulse of the vGRF during an SPJ in volleyball indirectly supported the previous study that indicated jumping height variations were not found after an internal focus intervention [31]. The fact that video feedback did not modify the impulse of the vGRF supported our hypothesis. The decreased value on the vGRF at the first peak and in the region near it (3.13–5.94%) and the loading rate to the first peak after the video feedback could be explained by conscious motor control that an internal focus is more related to body segment control [32]. The first peak was formed by the heel strike, which was in the braking phase that was a passive phase for participants. The decreased first peak of the vGRF and its loading rate represented a better initial braking control of participants during an SPJ. Many studies have demonstrated that the increase in the peak of the vGRF results in the rising of injury risk of lower limbs [33,34]. Hence, it is reasonable that video feedback can decrease knee injuries in a volleyball spike jump. Although the difference of the second peak of the vGRF was not found in this study, the vGRF value near the second peak region (19.83–21.97%) was still reduced compared with before video feedback. The second peak of the vGRF that still belonged to the braking phase formed by the transition to forefoot contact and the decrease in the region near the peak could be explained by conscious motor control as well. The third peak of the vGRF during the propulsion phase was not found to increase significantly whereas a larger vGRF was found compared with before in the last (fourth) phase of the propulsion phase (91.43–100%). It suggested that participants may have obtained more power in the last phase after video feedback. Overall, the vGRF results indicated that video feedback could decrease the risk of injury to lower limbs without hampering performance.

A larger initial contact angle and maximal flexion angle of the knee found in the current study after the video feedback represented more flexor posture during an SPJ landing, which could result from a greater internal focus caused by video feedback. Meanwhile, a larger flexion angle was found in the region near the maximal flexion angle from 31.17–73.19%, which also indicated video feedback affected the control during the middle phase of an SPJ. The maximal extension and flexion moment decreased in this study as well, which also suggested a smaller knee load in the sagittal plane. A flexor posture can decrease knee load during the stop-jump [35]; meanwhile, a large number of cadavers and in vivo studies have shown that the increase of the valgus load will directly increase the strain of the knee [4,5,36,37]. Therefore, we can infer that video feedback could decrease the knee injury risk in the stop-jump of volleyball.

The maximal abduction moment decreased in the current study. It has been shown that the knee abduction moment can cause the tibia to move forward and eventually result in the load increasing of the ACL several times [38]. The decreased maximal abduction moment in our study indicated a lower knee injury risk. An increase in the knee extension moment has been shown not to directly relate to a higher knee load [39] but the knee biomechanics literature overwhelmingly suggests that a knee extension moment can be used to predict the forward sheer force of the proximal tibia [38,40,41], which is related to knee injury risk. Further research has proven that during a stop-jump, the knee extension moment is proportional to the forward tibia shear force [35]. Therefore, a larger knee extension moment combined with a larger abduction moment on the tibia is more likely to cause acute knee injuries. A larger adduction angular velocity of the knee was observed in the current study in the region 49.07–62.64% after video feedback in which participants transferred from a braking phase to a propulsion phase, which was associated with power development. Therefore, the observed larger extension moment and abduction angular velocity indicated a higher knee injury risk before video feedback.

An increased maximal internal angular velocity and moment of the tibia were found in this study whereas a decreased maximal external moment of the tibia was found after the video intervention. The SPM results showed that the external rotation angular velocity decreased from 45.85–49.96% when the participant was in the braking phase. Tibial external rotation is related to ligament dominance in the landing, which has been described as a lower extremity motion pattern that is associated with injuries in landing [42]. The results indicated that video feedback contributed to knee injury prevention. The results showed that video feedback increased the external rotation moment during the phase from 85.55–95.06% that was the last phase of the take-off but the angular velocity could not be found to be increased therefore whether this change facilitated a take-off needs to be further explored.

The findings in the present study provided the implication that natural video feedback potentially decreased the knee injury risk in a volleyball spike jump without undermining performance. However, muscle activities are needed for further exploration in the future.

## 5. Conclusions

The observations from our study suggested that video feedback in a natural practice has the potential to improve knee biomechanics whilst not altering the performance in a volleyball spike jump. This indicated that the CAH theory extracted from the harsh laboratory settings are possibly not suitable in real competitions as players can balance their attention focus automatically. However, further research on relevant muscle activities should be focused on in the future.

## 6. Limitations

Lacking muscle activities is an important limitation in the present study. To date, specific studies on the effect of attention on muscle force production are relatively limited. Research has reported integrated electromyography (iEMG) was reduced under external attention condition [32] whereas coordination among muscle groups was not affected by attention focus [43]. Force generation requires holistic body coordination to a single outcome. The muscle activities are affected by consciousness sophisticatedly. Another limitation of the present study is that real injury cannot be measured directly so we concluded according to knee movements. The third limitation is that we only detected the immediate effect and the long-term effect must be considered in training.

## Figures and Tables

**Table 1:** The results of the vGRF.

Parameters	Pre	Post	T	P
First peak vGRF (BW)	2.71 ± 0.32	1.41 ± 0.21	7.618	0.001 *
Second peak vGRF (BW)	1.91 ± 0.14	1.72 ± 0.18	2.286	0.071
Third peak vGRF(BW)	1.54 ± 0.11	1.45 ± 0.17	1.369	0.213
First loading rate (BW/S)	81.16 ± 10.61	50.58 ± 23.79	4.205	0.004 *
Second loading rate (BW/S)	58.03 ± 4.37	57.31 ± 7.30	0.254	0.808
Impulse (BWs)	0.60 ± 0.07	0.53 ± 0.04	1.712	0.131

Note: * indicates a statistical difference (*p* < 0.05).

**Table 2:** The outcomes of knee kinematics and kinetics in three-movement planes.

Parameters	Pre	Post	T	P
Approaching velocity (m/s)	0.49 ± 0.07	0.46 ± 0.11	0.751	0.477
Initial contact flexion angle (°)	−46.23 ± 6.64	−51.53 ± 8.93	2.179	0.049 *
Maximal flexion angle (°)	−102.90 ± 12.36	−108.53 ± 8.15	3.242	0.006 *
Maximal flexion angular velocity (°/s)	−240.93 ± 26.62	−237.17 ± 27.67	0.848	0.413
Maximal extension angular velocity (°/s)	449.21 ± 59.88	435.85 ± 72.70	−0.073	0.943
Maximal extension moment (Nm/kg)	3.75 ± 0.48	3.51 ± 0.43	3.962	0.001 *
Maximal flexion moment (Nm/kg)	−1.86 ± 0.63	−1.19 ± 0.55	−3.711	0.002 *
Maximal flexion power (W/kg)	−20.46 ± 3.84	−20.52 ± 4.71	−0.677	0.518
Maximal extension power (W/kg)	20.36 ± 3.47	21.26 ± 1.60	0.249	0.811
Maximal abduction angle (°)	5.36 ± 1.29	6.08 ± 2.03	−1.238	0.304
Maximal adduction angle (°)	−3.81 ± 1.91	−3.64 ± 2.68	−0.353	0.747
Maximal abduction angular velocity (°/s)	385.99 ± 106.10	354.53 ± 103.38	0.989	0.340
Maximal adduction angular velocity (°/s)	−343.37 ± 52.92	−338.13 ± 69.47	0.307	0.763
Maximal abduction moment (Nm/kg)	0.55 ± 0.18	0.29 ± 0.05	3.069	0.037 *
Maximal adduction moment (Nm/kg)	−0.49 ± 0.07	−0.48 ± 0.12	−0.081	0.939
Maximal abduction power (w/kg)	0.37 ± 0.13	0.60 ± 0.17	−2.258	0.087
Maximal adduction power (w/kg)	−0.29 ± 0.10	−0.40 ± 0.14	1.166	0.308
Maximal external rotation angle (°)	9.57 ± 1.33	4.84 ± 4.46	2.610	0.121
Maximal internal rotation angle (°)	−3.51 ± 1.08	−6.09 ± 1.59	1.961	0.189
Maximal external rotation angular velocity (°/s)	101.15 ± 9.16	95.83 ± 37.29	0.288	0.785
Maximal internal rotation angular velocity (°/s)	−3.41 ± 5.23	−21.41 ± 10.82	5.209	0.003 *
Maximal external rotation moment (Nm/kg)	1.34 ± 0.31	1.08 ± 0.19	2.348	0.041 *
Maximal internal rotation moment (Nm/kg)	−0.24 ± 0.12	−0.37 ± 0.22	2.813	0.018 *
Maximal external rotation power (w/kg)	0.56 ± 0.55	0.88 ± 0.34	1.900	0.099
Maximal internal rotation power (w/kg)	−0.59 ± 0.14	−0.44 ± 0.22	−1.524	0.171

Note: * indicates a statistical difference (*p* < 0.05).

## Data Availability

Data are available on request due to restrictions on privacy. The data presented in this study may be available on request from the corresponding author and with authorization of funding origination.
